# Severe Aplastic Anemia following Acute Hepatitis from Toxic Liver Injury: Literature Review and Case Report of a Successful Outcome

**DOI:** 10.1155/2014/216570

**Published:** 2014-12-22

**Authors:** Kamran Qureshi, Usman Sarwar, Hicham Khallafi

**Affiliations:** ^1^Section of Gastroenterology and Hepatology, Division of Hepatology, Department of Medicine, Temple University School of Medicine, Temple University Health System, 3440 N Broad Street, Kresge Building West No. 209, Philadelphia, PA 19140, USA; ^2^Temple University Hospital, 3401 North Broad Street, Philadelphia, PA 19140, USA; ^3^Division of Gastroenterology and Hepatology, Department of Medicine, Case Western Reserve University School of Medicine, MetroHealth System, Medical Center, 2500 MetroHealth Drive, Cleveland, OH 44109, USA

## Abstract

Hepatitis associated aplastic anemia (HAAA) is a rare syndrome in which severe aplastic anemia (SAA) complicates the recovery of acute hepatitis (AH). HAAA is described to occur with AH caused by viral infections and also with idiopathic cases of AH and no clear etiology of liver injury. Clinically, AH can be mild to fulminant and transient to persistent and precedes the onset SAA. It is assumed that immunologic dysregulation following AH leads to the development of SAA. Several observations have been made to elucidate the immune mediated injury mechanisms, ensuing from liver injury and progressing to trigger bone marrow failure with the involvement of activated lymphocytes and severe T-cell imbalance. HAAA has a very poor outcome and often requires bone marrow transplant (BMT). The findings of immune related myeloid injury implied the use of immunosuppressive therapy (IST) and led to improved survival from HAAA. We report a case of young male who presented with AH resulting from the intake of muscle building protein supplements and anabolic steroids. The liver injury slowly resolved with supportive care and after 4 months of attack of AH, he developed SAA. He was treated with IST with successful outcome without the need for a BMT.

## 1. Introduction

Hematologic abnormalities are commonly seen in the patients with acute or chronic liver disease. These derangements are mostly due to nutritional deficiencies, concurrent autoimmune diseases, hypersplenism, or portal hypertension. Severe aplastic anemia (SAA) is defined as severe pancytopenia with at least two of the following abnormalities: an absolute neutrophil count (ANC) of <500/mm^3^, a platelet count of <20 × 10^3^/mm^3^, and a reticulocyte count of <60 × 10^3^/mm^3^ in the presence of bone marrow cellularity of <30% [1]. SAA can rarely complicate the course of acute hepatitis (AH) and presents as an acute bone marrow failure within a few weeks to months of an episode of acute liver injury [2]. A few studies have described the occurrence of SAA following 0.03–0.2% of cases of AH [3]. Looking at its prevalence from the hematological standpoint, 2–5% of cases of SAA in Western studies [4], 10% of adults, and as high as 25% of children with SAA in Asian studies have AH documented to be present prior to SAA [5]. This association is labelled as hepatitis associated aplastic anemia (HAAA) in literature and is considered one of the causes of secondary SAA in young population. SAA is mostly seen to occur in adolescent males and presents with the clinical picture of pancytopenia within 1 week to 6 months after an episode of clinical AH [6]. HAAA was first described in 1955 [7], and since then the syndrome has been well defined and several pathogenesis mechanisms have been suggested. It has been reported in association with viral hepatitis related to hepatitis A, B, C, and G infections. Also, Parvovirus, Epstein Barr virus (EBV), transfusion transmitted virus (TTV), and echovirus have been implicated as causative agents [8]. However, in most of the cases, no specific etiology of AH could be identified on clinical and serologic basis. Recently, a case of HAAA was reported in the literature and an anabolic steroid methasterone was linked to the development of transient cholestatic hepatitis and subsequently aplastic anemia [9]. Untreated HAAA has high mortality and survival of initially described cases was dismal [6]. Frequently, patient died from the complications of SAA and bone marrow transplant (BMT) was later used to treat HAAA. More recently, HAAA is being treated with immunosuppression and BMT is done only in cases of refractory SAA.

We illustrate the case of an adult male who was initially managed for a probable DILI and resultant AH in our hospital and whose clinical course of recovery from AH was complicated with development of SAA. With prompt identification and management, his HAAA was successfully treated with IST along with our hematology colleagues and patient recovered without needing a BMT. This review summarizes the literature on this rare and often fatal syndrome and suggests the extension of the spectrum of etiologic definition of HAAA.

## 2. Case Report

**Figure 1:**
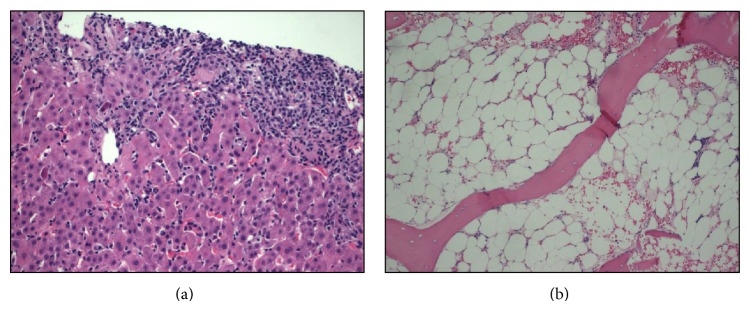
(a) Liver biopsy demonstrating features of active hepatitis, impressive inflammatory process involving both portal areas, and the lobules displaying also a pattern of sinusoidal lymphocytosis. (b) Bone marrow biopsy showing severe marrow hypocellularity.

**Figure 2:**
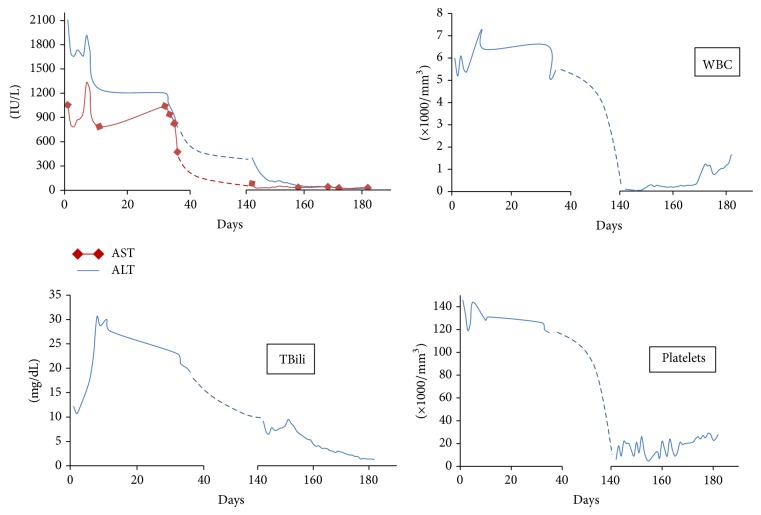
Graphical trends of the laboratory parameters of HAAA over six months.

We describe a case of a 26-year-old Hispanic male, who presented (Day 1) to his primary care physician (PCP) office after he noticed progressively worsening yellowish discoloration of his eyes and skin for 10 days' duration. In addition, he had noticed dark urine for 2-3 weeks and pale colored stools for 5–7 days. He complained of nausea, generalized fatigue, and malaise but did not have any abdominal pain, fever, chills, diarrhea, or any skin rash. He was noted to have diffuse jaundice, hepatomegaly, and mild epigastric tenderness on examination. The laboratory evaluation revealed abnormalities in liver panel, with total bilirubin (TBili) of 12.2 mg/dL, alkaline phosphatase (AlkP) 272 IU/dL, alanine aminotransferase (ALT), and aspartate aminotransferase (AST) of 2112 and 1055 IU/dL, respectively (Table 1). The complete blood count (CBC) and coagulation panel (INR) were normal at that time. He was admitted to our hospital where he underwent initial workup for painless jaundice. Upon initial evaluation by hepatology service, he informed us that he was originally from Puerto Rico and was living in the US for 17 years. He denied any history of significant illness as a child or any known history of liver disease in any family member. He denied any episodes of mental confusion and excessive sleepiness, as well as hematemesis, hematochezia, melena, or poor appetite. He also denied pruritus at any time and lower extremity edema or increased abdominal girth. He denied any recent sick contacts, animal exposure, or travel outside the US. He denied any history of incarceration, tattoos, or blood transfusions. He denied any history of tobacco use, illicit drug usage such as marijuana, cocaine, and heroin, or abuse of amphetamines. He reported drinking alcohol only on occasions and his last drink was approximately 7 months prior to this admission. However, he did report that he had been using over-the-counter anabolic steroids and a supplement from a vitamin store as a muscle-building high performance protein supplement (the ingredients are indicated in Table 3) on a daily basis for approximately 6 months. On examination, he appeared comfortable with diffuse jaundice and somewhat tender hepatomegaly. No clinical stigmata of advanced liver disease were identified on examination. The baseline serologic workup is shown in Table 2 which ruled out any infectious, autoimmune, or metabolic causes of his liver disease. The radiological workup with ultrasound, and a Magnetic Resonance Cholangiopancreatography did not reveal any biliary obstruction. He was suspected to have probable DILI with significant hyperbilirubinemia based on the negative etiologic workup. For further confirmation, he underwent a liver biopsy (Day 5) which revealed quite an impressive inflammatory process involving both portal areas and the lobules displaying also a pattern of sinusoidal lymphocytosis (Figure 1). The hepatocytic injury was identified and was more prominent in centrilobular (zone 3) location. Trichrome stain revealed only mild portal and periportal fibrosis and some perisinusoidal fibrosis especially in centrilobular location where the majority of the hepatocytic damage was identified along with mild collapse of the reticulin framework and likely was the result of hepatocytic dropout. Within the lobules, there was prominent “spotty necrosis,” highlighted with the PAS positive, diastase resistant stain, revealing macrophages loaded with phagocytic debris. Iron stores were not increased and immunostain for adenovirus, cytomegalovirus (CMV), herpes virus, and hepatitis B surface antigen were also negative on the histologic tissue. Iron stain was negative and copper stain showed no increased copper deposition in the hepatocytes. Based on the above findings, he was started on a short course of oral prednisone and ursodiol (Day 8) as the treatment for severe AH with cholestasis. His laboratory tests showed gradual improvement in hepatitis and subsequently he was discharged to be followed up as outpatient. He was seen in the clinic for monitoring (Day 35) and reported over all symptomatic improvement and he had started back his job as a residential painter. His follow-up monitoring laboratory testing 6 weeks later showed continued improvement in AH (Figure 2) while he stayed on low dose ursodiol.

On his next set of monitoring laboratory testing (Day 142), new onset severe pancytopenia (Table 1) was identified which prompted urgent hospital admission for evaluation and management of his pancytopenia. All viral etiologies of acute pancytopenia were ruled out by serologic analysis. He did not have any family history of AA. Extensive hematological workup was performed and all other causes of primary SAA were ruled (negative anti-CD55 and anti-CD59 antibodies, negative urinary collections for lead and arsenic, and negative flow cytometry). Peripheral blood smear analysis revealed severe neutropenia and normocytic anemia with frequent target cells, consistent with clinical history of liver disease along with severe thrombocytopenia. He underwent a bone marrow biopsy and flow cytometry analysis which showed severe hypocellular bone marrow (less than 5%) with dyserythropoiesis (Figure 1). The cytogenetic study showed normal karyotype. Immunostain with CD3 and PAX5 stains showed no involvement of lymphoma. CD34, TdT, and CD117 stains confirmed no significant increase of blasts. D31 and Factor-VIII stains show virtual absence of megakaryocytes. In view of the patient's age, gender, and his presentation with initial AH, the diagnosis of HAAA was made. The patient did not have any full siblings and in view of the absence of matched HLA siblings, the decision was made to immediately initiate IST. He initially received thymoglobulin (ATG) along with methylprednisolone treatment for a total of 5 days. He received prophylactic antimicrobials, in addition to filgrastim daily and platelet transfusions as needed to support his peripheral cell count. He responded to the induction therapy with the improvement in cell counts and significant reduction in his liver enzymes. Later, he was kept on cyclosporine (Cys) and prednisone was tapered off. This resulted in stable cell counts and partial recovery of bone marrow (Day 160). IST was continued as cyclosporine monotherapy and further improvement in cell counts was seen (Figure 2). He continues on cyclosporine with complete recovery of HAAA (Table 1) after 3 years of initial presentation.

## 3. Discussion

The unique aspect of our case is the etiology of AH, which has not been widely reported in the past as a specific cause of AH leading to HAAA. Hepatotoxicity in the form of cholestasis and hepatitis has been well described in the literature resulting from anabolic steroids and also occasionally with protein supplements [10, 11]. These agents are not specifically considered myelotoxic. Our patient was taking these over-the-counter products for more than 3 months prior to the initiation of symptoms and subsequent diagnosis of AH. After the diagnosis of AH and the cessation of those products, the hepatocellular injury pattern improved with ALT decreasing to > 50% in a month. Extensive evaluation was undertaken and it ruled out presence of any concomitant toxic, viral, autoimmune, or metabolic causes of AH (Table 3). Also, there were no other clinical risk factors, high risk behavior, sick contact, or a recent travel identified in this case which could contribute to unidentifiable cause of his AH. Based on this data and by using Roussel Uclaf Causality Assessment Method (RUCAM) [12], we calculated the score of 7, which suggested that those products are the “probable” cause of his liver injury and AH. We did not check for hepatitis E and G viruses, GB virus C, or TTV viruses which have been implicated as etiologic agents leading to HAAA. The clinical suspicion for these viral infections was low and also laboratory assays were not available for clinical use.

Our patient followed the typical stereotypic presentation of HAAA which most often develops in male adolescents or young men. Our patient showed evidence of bone marrow failure 4 months after the onset of AH. There is currently no clear determination of the duration of the onset of hepatitis and a diagnosis of HAAA; it varies from less than a year to less than 3 months [13, 14]. HAAA most often occurs in the recovery period after AH. In one study, AH completely resolved in only 60% of cases [15] while the rest of the patients had mild persistent hepatitis, as was the case with our patient. The typical hepatitis viruses including A, B, C, D, E, and G and other viruses such as Parvovirus B-19, CMV, Epstein-Barr virus, TTV, and non-A-E hepatitis virus have been implicated as a cause of AH and subsequent development of HAAA [16]. We screened our patient with all of the available serologic assays. Our clinical suspicion for the rare forms of viral hepatitis was low. The symptoms of hepatitis have been reported to be ranging from mild to fulminant liver failure requiring liver transplantation (LT). Our patient presented with insidious onset of cholestasis and liver injury typical for anabolic steroids hepatotoxicity [17, 18]. HAAA is reported to arise even after LT in up to 30% of children who underwent LT for non-A, non-B, and non-C hepatitis related liver failure [19] suggesting continuum of underlying pathogenic mechanism even after the curative treatment of inciting event. Our patient was given a short course of prednisone for treatment of drug induced liver injury based on the past experience [20], although it is widely believed to be ineffective in such drug induced liver injury. The clinical presentation of SAA after AH is variable and oftentimes it is diagnosed on routine laboratory testing as the new onset pancytopenia. The clinical symptoms of SAA include spontaneous bleeding (mucosal or cutaneous) related to thrombocytopenia, fatigue, and pallor caused by progressive anemia, fever, mucosal ulcerations, and infections secondary to neutropenia. Intracranial bleeding and severe sepsis are identified as the most common fatal complications of HAAA [6]. Our patient was lucky to be identified on the routine monitoring laboratory testing before he developed any complications of pancytopenia. He underwent an extensive workup to rule out primary aplastic anemia or other causes of acquired SAA. His bone marrow biopsy showing severe hypocellularity (<5%) and hematologic evaluation suggested SAA and the history of proceeding AH; in view of his age, gender, and timeline of events, HAAA was a strongly considered diagnosis. Curiously, the globulin levels were noted to be normal at the onset and gradually decreased to low levels in our patient. Severe hepatitis with features similar to autoimmune hepatitis has been reported in patient with common variable immunodeficiency (CVID) [21]. CVID is a syndrome which is characterized by various degrees of primary hypogammaglobulinemia and is frequently associated with autoimmune diseases [22]. Our patient did not have any history of recurrent sinopulmonary or gastrointestinal infections. While CVID remains an interesting differential in the diagnosis of our patient, clinical resolution of AH after avoiding the offending supplements and normalization of globulin levels after complete recovery of HAAA would favor a toxic etiology. CVID is diagnosed by excluding the causes of acquired immunodeficiency and that workup was not performed in our patient.

In the mechanistic studies of patients with HAAA, several immunological abnormalities have been described and a favorable response to IST has suggested immunologic dysregulation as the main pathogenic mechanism leading to HAAA. Patients with HAA were found to have a decreased ratio of CD4/CD8 cells in peripheral blood, which is associated with activated cytotoxic T cells and an increase in the proportion of CD8 cells that are HLA-DR positive [2, 23]. Activated CD8-positive lymphocytes have been implicated to be cytotoxic to myelopoietic cells in the bone marrow in patients with aplastic anemia [24]. In addition, interferon-gamma is found to be a marrow suppressing cytokine [25] and is secreted by activated T cells. Intense lymphocytic infiltrate is seen on histologic evaluation of AH from viral hepatitis and predominantly consists of T cells. CD-8 expressing Kupffer cells could be important mediators of HAAA [23]. The liver biopsy of our patient showed significant sinusoidal lymphocytosis. The time interval between the occurrence of hepatitis and the onset of bone marrow failure in HAAA suggests that the initial target organ of the immunological response is the liver [26]. A recent study suggested that T-lymphocytes clones are formed in the early stage of AH which recognize similar target antigens against both hepatocytes and myeloid cells. Subsequent selective expansion of the clones that are highly tropic to bone marrow could lead to HAAA [27].

The major curative options which are evaluated for treating severe HAAA are BMT and IST [28]. Supportive care is provided by prompt treatment and prophylaxis of possible infectious complications, antiviral prophylaxis of hepatitis B carriers, institution of hematopoietic growth factors, and blood products transfusion as needed [29]. Overall BMT has better survival (up to 82%) [8, 30] and a search for a Human Leucocyte Antigen identical sibling as a donor of bone marrow, to enable BMT to be undertaken promptly. Our patient did not have a matching family member. IST is an alternative first line treatment for a patient without an option of BMT [30] with a mean response rate of 70% [8]. Initial induction regimens when tested as ATG alone or Cys alone were associated with response rates of about 50% [31]. This led to the use of combination regimens of ATG, Cys, corticosteroids, and hematopoietic growth factors with the response rates of 75–80% [31]. Those patients who do not respond to the initial IST either can be managed by different IST regimens [32] or can be offered a matched unrelated donor, but overall prognosis is dismal is such cases [2, 33]. Successful treatment with IST is usually associated with rapid resolution of AH in patients with persistent hepatitis. Cytotoxic T-lymphocytes are thought to cause ongoing hepatic damage ATG and Cys may suppress those cells and improve hepatitis as well as bone marrow failure.

In conclusion, HAAA is a well-defined clinical syndrome in which an attack of hepatitis leads to bone marrow failure through immunologic mechanisms. Overall prognosis of unrecognized and thus untreated cases is very poor. HAAA mostly affects young male population who present with illness of viral or nonviral AH and later progress to SAA. Our report suggests extending the etiologic spectrum of HAAA to involve DILI as the inciting cause. Prompt identification and referral to BMT centers are imperative. We were able to identify our patient and coordinate his care of HAAA in a timely manner, which resulted in the recovery of liver injury and bone marrow failure with institution of IST. He is being followed in our clinic for over 3 years and has shown a successful recovery from HAAA following a toxic liver injury.

## Figures and Tables

**Table 1:** Laboratory test flow chart.

Lab	Day 1	Day 8	Day 35	Day 142	Day 160	Day 180	2 years	3 years
WBC	6		5.45	0.11	0.22	1.58	3.9	4.1
RBC	5.18		5.29	2.81	2.99	3.61	4.28	4.92
Hgb	15.7		15.2	8.4	8.7	10.8	13.7	14.9
Platelets	146		117	6	22	34	97	92
ANC	3.53		4.23	0.05	0.15	0.75	2.2	2.2
Bands				0.02	0.03	0.11		
INR	1		1.1		1.1			
Glucose	93	98	94	142	97	99	14	16
Blood urea	12	13	10	30	16	19		
Creatinine	0.88	0.85	0.86	0.78	1.03	0.91	0.99	0.97
Protein	7	5.9	4.6	4.6	5.7	5.9	6.9	7.1
Albumin	4.3	3.7	3.3	3.3	3.7	4.3	4.5	4.5
Globulin	2.7	2.2	1.3	1.0	2.0	2	2.4	2.6
T bilirubin	12.2	30.5	20	9.2	4.4	1.4	0.8	0.7
AlkP	272	231	319	119	234	126	130	114
ALT	2112	1747	922	381	43	17	31	24
AST	1055	1251	827	75	27	23	25	20

WBC: white blood count × 1000/mm^3^; RBC: red blood cells × million/mm^3^; Hgb: hemoglobin × g/dL; ANC: absolute neutrophil count × 1000/mm^3^; INR: international normalized ratio; AlkP: alkaline phosphatase IU/dL; ALT: alanine aminotransferase IU/dL; AST: aspartate aminotransferase IU/dL.

**Table 2:** Initial acute hepatitis workup.

Antinuclear antibodies	Negative	Ferritin	829
Antimitochondrial antibodies	Negative	Iron	173
Smooth muscle antibody	Negative	TIBC	337
Liver kidney microsomal antibody	Negative	% sat.	51
Cytokeratin antibody	Negative	IgG	414
Anti-Smith antibody	Negative	IgA	44
Hepatitis A IgM	Nonreactive	IgM	63
Hepatitis B core IgM	Nonreactive	Alpha1AT	327
Hepatitis B surface antigen	Nonreactive	AFP tumor marker	22.8
Hepatitis C IgG antibody	Nonreactive	Ceruloplasmin	35
HIV 1 & 2 antibody	Nonreactive	Adenovirus IgG antibody	1.5
CMV IgG antibody	Positive	Adenovirus IgM antibody	0.15
CMV IgM antibody	Negative	Adenovirus PCR quantitative	No DNA detected
CMV PCR quantitative	<100	Ethanol level	<10
Herpes 1 IgG antibody	Negative	Ur amphetamine screen	Negative
Herpes 2 IgG antibody	Negative	Ur barbiturate screen	Negative
HSV IgM antibody	Negative	Ur benzodiazepine screen	Negative
HSV PCR qualitative	Not detected	Ur cocaine screen	Negative
EBV IgG antibody	Positive	Ur methadone screen	Negative
EBV IgM antibody	Negative	Ur opiate screen	Negative
EBV ultraquantitative	<100	Ur PCP screen	Negative
Parvovirus B19 IgG antibody	Positive	Ur THC screen	Negative
Parvovirus B19 IgM antibody	Negative	Ur Tricyclics screen	Negative
Parvovirus B19 DNA PCR	Not detected	Hemochromatosis mutation	Negative

**Table 3:** Ingredient description of the used muscle building supplement.

Amount per serving		

Calories	140	
Calories from fat	35	
Total fat	3.00 g	
Saturated fat	3.00 g	
Cholesterol	5.00 mg	
Sodium	230.00 mg	
Potassium	490.00 mg	
Total carbohydrate	2.00 g	
Dietary fiber	0.00 g	
Sugars	0.00 g	
Protein	20.00 g	
Phosphorus	490.00 mg	
Calcium	670.00 mg	
Iron	3.50 mg	
Alanine	4230.00 mg	
Arginine	7040.00 mg	
Aspartic acid	11130.00 mg	
Cysteine	1250.00 mg	
Glutamine (as glutamic acid)	21710.00 mg	
Glycine	3830.00 mg	
Histidine	2600.00 mg	
Isoleucine	5950.00 mg	
Leucine	7650.00 mg	
Lysine	6500.00 mg	
Methionine	1380.00 mg	
Phenylalanine	5100.00 mg	
Proline	5430.00 mg	
Serine	5180.00 mg	
Threonine	3890.00 mg	
Tryptophan	1280.00 mg	
Tyrosine	3860.00 mg	
Valine	6010.00 mg	
Total amino acids	104020.00 mg	

Total fat	65 g	80 g
Sat. fat	20 g	25 g
Cholesterol	300 mg	300 mg
Sodium	2400 mg	2400 mg
Total carbohydrate	300 g	375 g
Dietary fiber	25 g	30 g

*Other Ingredients*. Sustained release amino acid enhanced protein matrix (whey peptides, whey protein concentrate, Supro brand and regular brand soy protein isolate, branched chain amino acid blend (*L*-isoleucine, *L*-leucine, valine (as *L*-valine))), Lipobolic & trade; advanced lipid complex (evening primrose oil (*Oenothera biennis*), conjugated linoleic acid (CLA) (80%), medium chain triglycerides, flax seed powder, borage seed oil powder, and omega-3 complex), natural and artificial flavors, stearic acid, gum blend (carrageenan, xanthan gum, and cellulose gum), beet color, silica, lecithin, malic acid, acesulfame potassium, sucralose, and citric acid		
